# Epididymal Adenomatoid Tumor: A Very Rare Paratesticular Tumor of Childhood

**DOI:** 10.1155/2016/9539378

**Published:** 2016-11-27

**Authors:** Ioannis Patoulias, Christos Kaselas, Dimitrios Patoulias, Constantine Theocharides, Maria Kalogirou, Konstantinos Farmakis, Thomas Feidantsis

**Affiliations:** ^1^First Department of Pediatric Surgery, Aristotle University of Thessaloniki, G. H. G. Gennimatas, 41 Ethnikis Aminis Street, 54635 Thessaloniki, Greece; ^2^Department of Pathology, G. H. G. Gennimatas, Thessaloniki, Greece

## Abstract

Adenomatoid tumor is an uncommon benign mesothelial neoplasm, usually localized in the epididymis. It is the most common paratesticular tumor of middle-aged patients (average age of clinical presentation: 36 years). However, these tumors in pediatric and pubertal patients are extremely rare. Due to their rarity, we present a case of adenomatoid tumor of the tail of the epididymis in a 16-year-old patient. After systematic research of the current literature, we did not find another case report of epididymal adenomatoid tumor in a male patient aged 16 years old or less. This notice and our concern, as well, about the patient's surveillance protocol during the postoperative period were the motive for this case study.

## 1. Introduction

Adenomatoid tumor of the male genital tract is a nonhormone dependent tumor of mesothelial origin that is usually localized in the epididymis [1]. Paratesticular tumors account for less than 5% of all intrascrotal masses. Adenomatoid tumors are the most common paratesticular neoplasms, comprising about 30% of them [2]. Beccia et al. [3] reported that 256 epididymal tumors of 341 in total (75%) were benign. Among those epididymal tumors, adenomatoid tumor (73%), leiomyoma (11%), and papillary cystadenoma (9%) were the most frequent. The remaining benign entities (7%) included angioma, lipoma, dermoid cysts, fibroma, hamartoma, teratoma, and cholesteatoma.

According to its histological characteristics, adenomatoid tumor can be divided into 3 subtypes: tubular, angiomatoid, and plexiform. Amin and Parwani discriminate 4 kinds of adenomatoid tumors: adenoid or tubular glandular, angiomatoid, solid, and cystic or any transitional form of them [4]. Although various theories about their histogenesis have been formulated (mesothelial, endothelial, Müllerian, and mesonephric origin), the hypothesis of their mesothelial origin prevails, also supported by the electron microscopy study [5]. Major microscopic features include fibrous stroma and vacuolated epithelial cells. Vacuoles may vary in size; sometimes they occupy most of the cell's cytoplasm [2]. Nuclear atypia and local invasive behavior have sometimes been noticed, especially in tumors in the head of epididymis [1, 4].

After systematic research of the current literature, we did not find another published case report of epididymal adenomatoid tumor in a patient 16 years old or less. This notice and our concern, as well, about the appropriate patient's surveillance protocol during the postoperative period were the motive for this case study.

## 2. Case Report

A 16-year-old male with free medical history presented as an outpatient requesting for consultation regarding a small lump that he palpated two months ago in his right hemiscrotum during self-examination. He did not report any trauma or inflammation of the area neither at the referred period nor in the past. Except for mild discomfort in the hemiscrotum during exercise that subsided after its discontinuation, no other symptoms were reported. Importantly, the size of the lump did not change significantly during this two-month period.

Physical examination revealed a small, round, hazelnut-sized, painless mass in the right hemiscrotum, localized in the tail of the epididymis. No other pathological signs were detected at the rest of the scrotum, testicles, or groin.

**Figure 1:**
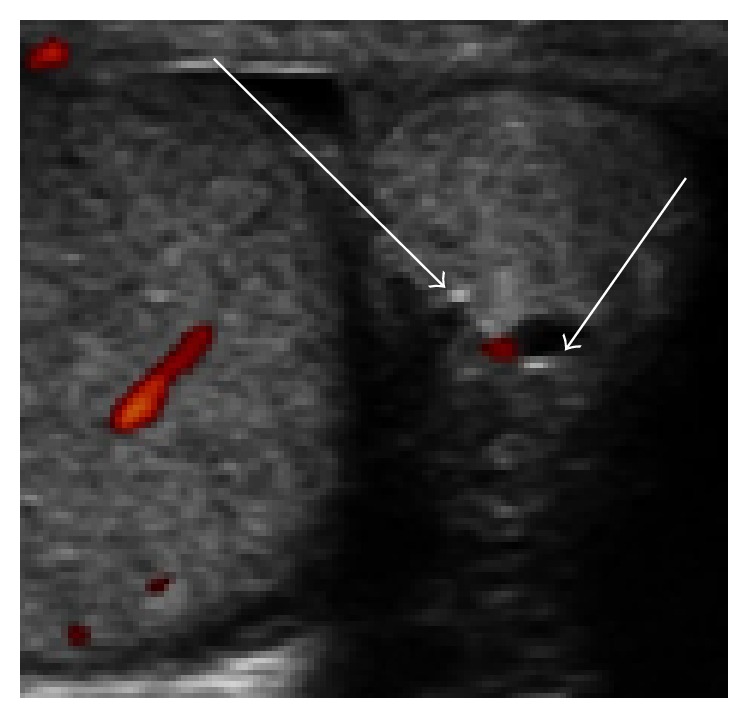
Solid hypoperfused, hyperechoic, 1.3 × 1.1 cm in size mass localized at the tail of the epididymis. Notice the 2 small hypoechoic lesions inside the mass.

U/S examination of the scrotum documented the presence of a solid hypoperfused, hyperechoic, well-demarcated, without invasive behavior mass, localized at the tail of the epididymis, 1.3 × 1.1 cm in size, and including 2 small hypoechoic lesions inside the mass (arrows, Figure 1).

**Figure 2:**
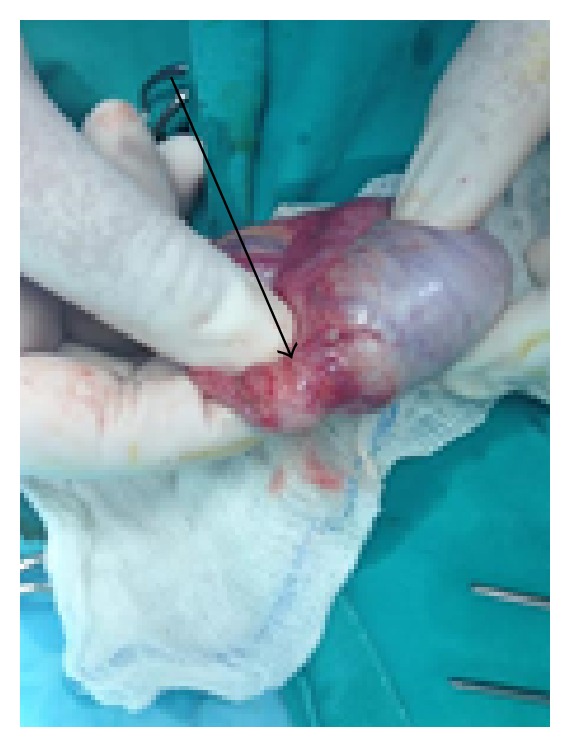
Yellowish uncapsulated mass (arrow) with maximum diameter of 13 mm located next to the tail of the epididymis.

The typical preoperative laboratory examination (blood routine and coagulation profile) and the values of the specific tumor markers AFP, LDH, CEA, and b-HCG were normal. Surgical treatment was decided and a right scrotal exploration was performed. Macroscopic examination revealed a yellowish uncapsulated mass with maximum diameter of 13 mm located next to the tail of the epididymis (Figure 2).

After meticulous dissection, the mass was totally dissociated from the epididymis and was excised en-block without any damage to adjacent structures.

**Figure 3:**
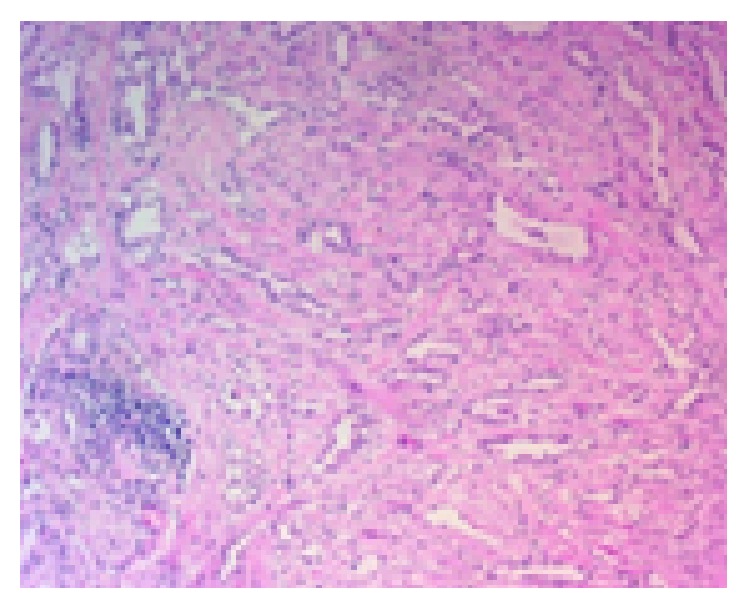
Adenomatoid tumor. Multiple irregular spaces (vacuolated cytoplasm) coated by a layer of flat or cuboidal epithelial cells and surrounded by collagenous stroma and muscle fibers (H-E 10x).

Histological examination of the mass revealed the presence of cuboidal epithelial cells in tubular clusters into a fibrous stroma (Figure 3). Immunohistochemical evaluation was positive for tumor markers HMBE1 and calretinin, which documented the diagnosis of adenomatoid tumor and its mesothelial origin.

Postoperative course was uneventful. After a 24-month follow-up period, the patient remains asymptomatic without signs of recurrence.

## 3. Discussion

Adenomatoid tumors are the most common tumors of middle-aged patients; average age of clinical presentation is 36 years [6]. After systematic research of the relevant literature, we did not find another published case of epididymal adenomatoid tumor in a patient younger than 16 years old [1, 2, 6–12]. Guo et al. [13] reported a case of adenomatoid tumor of tunica albuginea in a 12-year-old boy.

Adenomatoid tumors are usually incidental findings. Sometimes patients report mild discomfort or pain, mainly during exercise. Rarely, adenomatoid tumors can present as posttraumatic acute scrotum. Usually, there is no correlation between the tumor and previous scrotal inflammation or trauma. Differential diagnosis includes all possible testicular and paratesticular masses as well as other scrotal abnormalities such as lipoma, sarcoma, metastatic tumor, granuloma, and hematoma of the spermatic cord [14].

During the diagnostic approach, pediatric surgeon should emphasize on two major issues: the possibility of existence of subject malignancy and the feasibility of total and safe excision of the tumor. In older patients, when intraparenchymal involvement is noticed, differential diagnosis should necessarily include seminoma [15]. Ultrasonography, as the primary imaging modality, plays a significant role in preoperative diagnosis [4]. Sometimes local invasive behavior has been noticed, especially in tumors in the head of epididymis [1, 4]. Via ultrasonography, it is feasible to mark the boundaries of these tumors. In our case, preoperative imaging study showed a well-demarcated mass, without evidence of unclear boundaries or invasion into the adjacent structures. Thus, after evaluation of ultrasonographic findings, we assessed that MRI was not necessary.

However, MRI should be performed when ultrasonographic findings are unclear concerning tumor's boundaries and its local invasive behavior or in those cases that the mass arises from the tunica albuginea. It should be noted that 14% of adenomatoid tumors arise from the tunica albuginea. In those cases, MRI can distinguish the separating margin of the mass from the testicular parenchyma [13]. FNA is not recommended in general due to the possibility of malignancy [13].

If there is a strong evidence of malignancy based on the preoperative findings, excision of an extratesticular tumor is conducted after inguinal approach. Otherwise, approach can be scrotal. Mass enucleation without any damage to the adjacent structures is the basis of the treatment. If dissection of the mass from the adjacent structures is difficult, intraoperative biopsy should be conducted, which—in case of an adenomatoid tumor—leaves no doubt about its benign nature. In case of a mass with invasive behavior or documented malignancy, the need of complete or partial epididymectomy should be evaluated [1].

Microscopic features of adenomatoid tumor are characterized by three basic patterns: tubules, cords, and nests with cuboidal epithelium and amphophilic, eosinophilic, or vacuolated cytoplasm. Stroma is fibrous and occasionally hyalinized [15]. In our case, histological examination revealed the presence of multiple irregular spaces (vacuolated cytoplasm) coated by a layer of flat or cuboidal epithelial cells and surrounded by collagenous stroma and muscle fibers.

Immunohistochemically, an adenomatoid tumor is positive for markers, such as CK (AE1/AE3) ΕMΑ, Cam5.2, CK 5/6, CK7, calretinin, vimentin, WT1, and HBME-1. Other tumor markers, such as AFP, LDH, CEA, and b-HCG, when measured, are negative, being substantial for the exclusion of malignancy [4, 7, 8, 16, 17].

The mesothelial origin of adenomatoid tumor is confirmed by the identification of calretinin, which shows high sensitivity regarding identification of mesothelial cells. Calretinin is a calcium binding protein of the S-100 protein family that is expressed both in cytoplasm and in nucleus. Because of its expression in malignant tumors, absence of calretinin excludes the presence of malignancy [13]. Calretinin was positive in our case, documenting the mesothelial origin of the tumor. Two other mesothelial related markers, CK 5/6 and WT1, are also helpful both in diagnosis and in differential diagnosis from nonmesothelial lesions, even in cases of an infracted tumor [15].

Total tumor resection is considered to be curative as there are no reported cases of recurrence [13]. Scope of further exploration and application in clinical practice are the results of the recent study conducted by Hassan et al. [18]. In this report a spectrum of neoplastic lesions found in a patient with CADASIL syndrome is described. The male patient, 62 years old, had multiple neoplastic lesions that were observed during autopsy. He presented with a history of several neurological manifestations, including gait disturbance and frequent convulsive attacks. CADASIL syndrome was diagnosed, with identification of the Notch3 Arg133Cys mutation. The patient eventually developed hemiplegia and died due to systemic convulsions. Multiple neoplastic lesions—characteristic of CADASIL syndrome—such as carcinoid tumor let and diffuse idiopathic pulmonary neuroendocrine cell hyperplasia (DIPNECH) in the lungs, renal cell carcinoma (RCC), prostatic adenocarcinoma (ADC), and adenomatoid tumor of the epididymis that coexisted in this patient, as the autopsy revealed. Is there an incidental finding or it is a different clinical manifestation of the same pathogenesis? Further reports may confirm or not the real correlation.

Finally, we are concerned about the necessity of annual surveillance of the patient, which could consist of clinical examination, U/S examination of the upper and lower abdomen and the scrotum, chest X-ray, and specific tumor markers (AFP, LDH, CEA, and b-HCG).

## References

[1] Maestro M. A., Gonzalez R. T., Dorrego J. M. A., De la Peña Barthel J., De Serrano M. N. M. (2009). denometoid tumors of the epididymis and testicle: report of 9 cases and bibliographic review. *Archivos Españoles de Urología*.

[2] Kontos S., Fokitis I., Karakosta A. (2008). Adenomatoid tumor of epididymis: a case report. *Cases Journal*.

[3] Beccia D. J., Krane R. J., Olsson C. A. (1976). Clinical management of non-testicular intrascrotal tumors. *The Journal of Urology*.

[4] Amin W., Parwani A. V. (2009). Adenomatoid tumor of testis. *Clinical Medicine: Pathology*.

[5] Nistal M., Contreras F., Paniagua R. (1978). Adenomatoid tumour of the epididymis: histochemical and ultrastructural study of 2 cases. *British Journal of Urology*.

[6] Gupta A., Livingston M., Singh R., Tansey D., Solomon L. (2013). Infarcted adenomatoid tumour of epididymis: a rare case report. *Case Reports in Urology*.

[7] Venyo A. K., Baiden-Amissah K. (2011). Intra-scrotal adenomatoid tumour referred as an extra testis: a case report and review of the literature. *Urology*.

[8] Sangoi A. R., McKenney J. K., Schwartz E. J., Rouse R. V., Longacre T. A. (2009). Adenomatoid tumors of the female and male genital tracts: a clinicopathological and immunohistochemical study of 44 cases. *Modern Pathology*.

[9] Carranza A., Córdoba J. C., Sánchez J. M. (2010). Tumor adenomatoide paratesticular: una serie de nueve casos. *Actas Urológicas Españolas*.

[10] Damle R. P., Suryawanshi K. H., Dravid N. V. (2014). Adenomatoid tumor of epididymis—a case report. *International Journal of Health Sciences and Research*.

[11] Kojić D., Vukotić V., Boričić I., Babić U., Kapetanović S., Stavrić T. (2014). Evaluation of indolent epididymal mass—adenomatoid tumor of the epididymis. *Archives of Biological Sciences*.

[12] Chen D., Yu Z., Ni L. (2014). Adenomatoid tumors of the testis: a report of two cases and review of the literature. *Oncology Letters*.

[13] Guo K., Tian R., Liu L., Du C., Li F., Wang H. (2015). Adenomatoid tumor of the tunica albuginea in a boy: a case report and literature review. *Case Reports in Urology*.

[14] Cassidy F. H., Ishioka K. M., McMahon C. J. (2010). MR imaging of scrotal tumors and pseudotumors. *Radiographics*.

[15] Amin M. B. (2005). Selected other problematic testicular and paratesticular lesions: rete testis neoplasms and pseudotumors, mesothelial lesions and secondary tumors. *Modern Pathology*.

[16] Hes O., Perez-Montiel D. M., Cabrero I. A. (2003). Thread-like bridging strands: a morphologic feature present in all adenomatoid tumors. *Annals of Diagnostic Pathology*.

[17] Chandrasekar P., Tiwari A., Potluri B. (2003). Adenomatoid tumours of the male genital tract. *European Urology Supplements*.

[18] Hassan W. A., Udaka N., Ueda A., Ando Y., Ito T. (2015). Neoplastic lesions in CADASIL syndrome: report of an autopsied Japanese case. *International Journal of Clinical and Experimental Pathology*.

